# Prokaryote communities associated with different types of tissue formed and substrates inhabited by *Serpula lacrymans*

**DOI:** 10.1111/1758-2229.13191

**Published:** 2023-10-03

**Authors:** Julia Embacher, Susanne Zeilinger, Martin Kirchmair, Sigrid Neuhauser

**Affiliations:** ^1^ Institute of Microbiology, Universität Innsbruck Innsbruck Austria

## Abstract

The basidiomycete *Serpula lacrymans* is responsible for major timber devastation in houses. Basidiomycetes are known to harbour a diverse but poorly understood microbial community of bacteria, archaea, yeasts and filamentous fungi. In this study, we used amplicon‐sequencing to analyse the abundance and composition of prokaryotic communities associated with fruiting bodies of *S. lacrymans* and compared them to communities of surrounding material to access the ‘background’ community structure. Our findings indicate that bacterial genera cluster depended on sample type and that the main driver for microbial diversity is specimen, followed by sample origin. The most abundant bacterial phylum identified in the fruiting bodies was Pseudomonadota, followed by Actinomycetota and Bacteroidota. The prokaryote community of the mycelium was dominated by Actinomycetota, Halobacterota and Pseudomonadota. Actinomycetota was the most abundant phylum in both environment samples (infested timber and underground scree), followed by Bacillota in wood and Pseudomonadota in underground samples. *Nocardioides*, *Pseudomonas*, *Pseudonochardia*, *Streptomyces* and *Rubrobacter* spp. were among others found to comprise the core microbiome of *S. lacrymans* basidiocarps. This research contributes to the understanding of the holobiont *S. lacrymans* and gives hints to potential bacterial phyla important for its development and lifestyle.

## Graphical abstract

The dry rot fungus *Serpula lacrymans* mycobiome is mainly influenced by fungal tissue type followed by sample origin. Hence the composition of the prokaryote community associated with the fungus is distinct depending on sample, that is, the clusters formed by the community differ depending on whether they are associated with *S. lacrymans* tissues (e.g., fruiting body or mycelium) or with woody or non‐woody substrates.

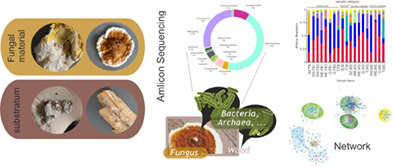

## INTRODUCTION

Structure and diversity of prokaryotic communities associated with fungi are increasingly studied (Bahram et al., 2018; Benucci & Bonito, 2016; Cullings et al., 2020; Liu et al., 2018; Tarkka et al., 2018). Inter‐kingdom interactions between fungi and prokaryotes co‐occurring in wood have been documented, and they cover the full range of symbiotic interactions from mutualism to antagonistic behaviour (Boddy et al., 2007; Boer et al., 2005; Clausen, 1996; Greaves, 1971; Johnston et al., 2016; Tornberg et al., 2003; Yurkov et al., 2012). Microorganisms that coexist in the same environment affect each other on various levels (e.g., metabolically or physiologically); hence, they establish a selective environment within which all partners are co‐ and interdependently developing. Examples of specialized interactions are bacteria which can promote hyphal growth (Q. Li et al., 2016; Oh et al., 2018; Schulz‐Bohm et al., 2017), or bacteria which induce fungal metabolite production (Tauber et al., 2018; Vahdatzadeh et al., 2015). However, there is also competition between prokaryotes and fungi for nutrients (Folman et al., 2008) and even mycophagy may occur, where prokaryotes feed on living (mycoparasitism) or dead (saprotrophy) fungal material.

Wood‐destroying fungi are responsible for substantial economic damage, especially in buildings. Their ecology and interaction with other microorganisms in the indoor environment, however, is so far not well understood. Because they destabilize wooden structures of buildings, fungi like *Coniophora puteana*, *Antrodia* spp. and *Serpula lacrymans* need to be removed from construction timber or—in case of *S. lacrymans*—also from the surrounding masonry via costly restoration procedures (Deutsches Institut für Normung (2020) or Austrian Standards (2015)). The dry rot fungus *S. lacrymans* is specialized on the degradation of coniferous timber and is characterized by rapid spread and wood degradation. The latter is predominantly affected by temperature and moisture as well as the durability of the wood material (Kržišnik et al., 2018). Because of the economic importance (Hyde et al., 2018) and the high effort that is needed to treat buildings in which *S. lacrymans* has been found, its interaction with prokaryotes has been explored to better understand the dynamics of dry rot (Embacher et al., 2021). Wood colonization by *S. lacrymans* can be supported by bacteria that are modifying wood structure and permeability using biopolymer degrading enzymes (e.g., cellulases and pectinases) that pre‐digest the structure of cellulose‐microfibrils in timber (Boutelje & Bravery, 1968; Rehbein et al., 2013). Wood has a high carbon content, but it is relatively poor in biologically available nitrogen; therefore, an increase in nitrogen caused by nitrogen‐fixing bacteria in exchange for carbon has been discussed as beneficial interaction between bacteria and fungi (Cowling & Merrill, 2011; Purahong et al., 2016; Tláskal et al., 2021). A relatively neutral and unspecific type of interaction is represented by Acidobacteriota, Bacteroidota and Actinomycetota among others that act as decomposers and that follow on the wood substrate to use the remaining cell wall components once *S. lacrymans* has degraded the cellulose (Berlemont & Martiny, 2013; Embacher et al., 2023; Tláskal & Baldrian, 2021). Some Pseudomonadota and other prokaryotes are able to obtain nutrients via mycophagy, which from the fungal viewpoint, is the negative end of interactions (Tláskal & Baldrian, 2021). Bacteria belonging to the genera *Bacillus*, *Pseudomonas*, *Alcaligenes*, *Serratia, Streptomyces*, *Erwinia* and *Enterobacteria*, among others, have been shown to play a role in the deterioration of building timber (Boutelje & Bravery, 1968; Dutkiewicz et al., 1992; Gaylarde & Morton, 1999; Line, 2003; Rogers & Baecker, 1991; Wójcik‐Fatla et al., 2022).

The aim of this study was to assess the biodiversity of prokaryotes associated with *S. lacrymans* with culture‐independent methods. We used amplicon sequencing to reveal the bacterial community composition in fruiting bodies and mycelial mats of *S. lacrymans* as well as wood and other substrates associated with the dry rot fungus. Differences in the prokaryotic biodiversity between and within samples were analysed. We aimed to test if (i) the structure of the fruiting bodies' bacterial communities is dissimilar to that of the surrounding wood‐ or mineral substrate, but more similar to that of the mycelium; and if (ii) there are any differences in community composition compared to our previous results from a culture‐based approach. The obtained results revealed a relatively small group of core operational taxonomic units (OTUs) associated with *S. lacrymans* fruiting bodies but characteristic prokaryote taxa associated with each fungal tissue type.

## EXPERIMENTAL PROCEDURES

### Sample collection and processing

**FIGURE 1:**
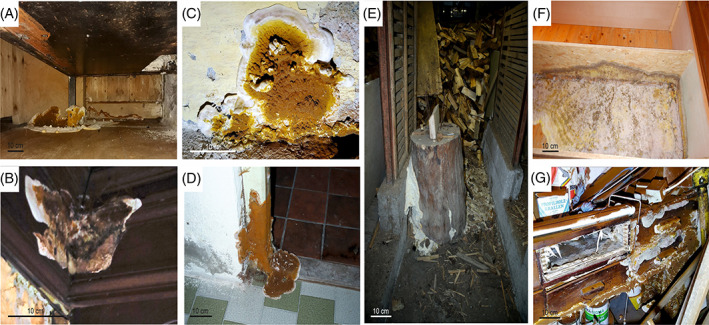
Fruiting bodies of *Serpula lacrymans* from distinct sampling points and examples for infestation of timber. (A) Zirl, (B) Reith/Alpbachtal, (C) Wolfsegg/Hausruck, (D) Grins and (E) Tarrenz. (F) Mycelium mat of *S. lacrymans*, (F) and (G) destruction caused by *S. lacrymans*.

Eight fruiting bodies, two mycelium mats and nine substrate samples (five wood and four mineral construction material such as plaster, brick walls and concrete) were collected from five infested houses in Austria (Table 1, Figures 1 and S1). It was not possible to sample mycelium mats at all sampling points; nonetheless, to complete the data set, we included the information from two mycelia mats.

**TABLE 1:** Sampling information.

Sampling point	Class				Sequence read archive no.
	Fruiting body	Mycelium mats	Wooden substrate	Mineral substrate	
Zirl (SZ)	1		1		SAMN30726529, SAMN30726530
Reith im Alpbachtal (SM)	2		1	1	SAMN30726531, SAMN30726532, SAMN30726533, SAMN30726543
Wolfsegg am Hausruck (WE)	2		1	1	SAMN30726534, SAMN30726535, SAMN30726536, SAMN30726537
Grins (G)	2	1	1	1	SAMN30726538, SAMN30726539, SAMN30726540, SAMN30726541, SAMN30726542
Tarrenz (T)	1	1	1	1	SAMN30726544, SAMN30726545, SAMN30726546, SAMN30726547

The samples were collected and transferred into fresh zip‐loc plastic bags. While wearing sterile gloves, the material was photo documented, small parts of the fruiting bodies and mycelium mats were cut out with a sterile scalpel, and transferred into 1.5 mL tubes filled with a sterile sphere matrix (0.2 g of glass beads, Ø 2 mm). Substrate material was transferred analogously into bead matrix tubes using a sterilized spoon, tweezers or a scalpel. The samples were stored at −20°C until further processing.

### DNA extraction and sequencing

Total genomic DNA was extracted from the sampled material using the Dneasy® PowerSoil® Pro Kit (Qiagen) according to the manufacturer's instructions with noted alterations. Briefly, samples stored in the tubes containing the sphere matrix (1.5 mL tubes with 0.2 g of glass beads with 2 mm diameter) were frozen with liquid nitrogen and homogenized twice using FastPrep‐24 5G (MP Biomedicals) at 6 m s^−1^ for 30 s (two cycles, 300 s pause).

Cells were treated with 800 μL CD1 buffer mixed with 10 μL Proteinase K solution (10 μg mL^−1^) and heated at 60°C for 10 min after mixing. The tubes were horizontally secured on a Vortex adapter and vortexed for 10 min at maximum speed. Further steps were executed according to the kit's instructions. In the last step, DNA was eluted from the columns using 50 μL of solution C6 (10 mM Tris). Three replicate extractions per sample were performed and the resulting DNA from all three extractions was pooled to represent the whole variability in our molecular fingerprint.

Sequencing was outsourced to Novogene Co., Ltd. The 16S rRNA gene variable regions V3–V4 were amplified using bacterial primers 341F (5′‐CCTAYGGGRBGCASCAG‐3′) and 806R (5′‐GGACTACNNGGGTATCTAAT‐3′). The samples were sequenced using the Illumina NovaSeq 6000 platform in rapid‐mode paired‐end 250 bp (PE250). The sequences obtained from each sample were submitted to Sequence Read Archive and are available under BioProject PRJNA878418 (accession numbers SAMN30726529 to SAMN30726547).

### Statistics and analysis of sequencing data

Novogene Co., Ltd. was commissioned for sequencing data quality control and further metagenomic and statistical analysis, including α‐ and β‐diversity. Our data were hence obtained using Novogene standard procedures that are briefly described below (for details see Supporting Information).

#### Sequencing data processing

Paired‐end reads were assigned to samples based on their unique barcodes and truncated by cutting off the barcode and primer sequences. They were merged using FLASH (V1.2.7; Magoč & Salzberg, 2011; merged raw reads 30k). Quality filtering of the raw tags followed the Qiime (V1.7.0; Caporaso et al., 2010) quality control process. The tags were compared with the reference database SILVA138 (Quast et al., 2013) using UCHIME (Edgar et al., 2011) for chimera detection and removal.

#### OTU clustering and taxonomic annotation

Sequences analysis was performed by Uparse (v7.0.1090; Edgar, 2013). Sequences with ≥97% similarity were assigned to the same OTUs. Each representative sequence was compared against the SSUrRNA database of SILVA138 (Q. Wang et al., 2007) for species annotation at each taxonomic rank (threshold: 0.8–1; kingdom, phylum, class, order, family, genus and species) using Qiime (version 1.7.0, method Mothur). To obtain the phylogenetic relationship of all OTUs of the representative sequences Muscle (version 3.8.31; Edgar, 2004) was used. OTU abundance was normalized against the sample with the least sequences. Normalized data were used for subsequent analysis of alpha and beta diversity.

#### Alpha diversity

Alpha diversity was assessed through community richness indices (Chao1 and ACE) and community diversity indices (Shannon and Simpson). Sequencing depth was evaluated using (the Good's coverage) and an index of phylogenetic diversity (PD_whole_tree). All indices were calculated with QIIME (version 1.7.0) and visualized with R software (version 2.15.3 by R Core Team (2018)).

#### Beta diversity

Beta diversity of weighted and unweighted unifrac‐distances was calculated by QIIME software (version 1.7.0). Cluster analysis was preceded by principal component analysis. FactoMineR (Lê et al., 2008) and ggplot2 (Hadley, 2016) package in R software (version 2.15.3) were used to reduce the dimension of the original variables. Principal coordinate analysis (PCoA) visualization was realized with WGCNA package (Langfelder & Horvath, 2008), stat packages and ggplot2 package in R software (version 2.15.3). Anosim, MRPP and Adonis were performed by R software (Vegan package: anosim function, mrpp function and adonis function; Jari et al., 2013). AMOVA was calculated by mothur (Schloss et al., 2009) using amova function. *T* test and drawing were conducted by R software.

### Network analysis

Networks were constructed based on 16S rRNA gene sequence data. The molecular ecological network analysis (MENA) pipeline (Deng et al., 2012) was used to analyse the networks. In brief, there were four main steps for network constructions: (i) original data collection (OTU table), (ii) data standardization (with centred log‐ratio transformation), (iii) pairwise correlation/similarity estimation and (iv) adjacent matrix formatting based on a random matrix theory method (Zhou et al., 2010). Default settings for data preparation and random matrix theory settings were used with noted alteration: the network was constructed using the OTUs present in at least 14 samples to increase the sensitivity of the analysis. To construct the network 0.76 was used as cutoff. ‘Greedy modularity optimization’ was the method for module separation. Indices to evaluate the features of the nodes were as follows: (i) degree—a node with higher degree means that it is highly connected with other nodes (i.e., high degree = strong relationship with others); (ii) betweenness centrality (BC) = among‐module connectivity (Pi; the parameter was used to indicate nodes connecting modules [connectors], Pi > 0.62); (iii) closeness centrality (CC) = within‐module connectivity (Zi), referring to highly connected nodes within modules (module hubs, Zi > 2.5); (iv) important nodes to both the network and its own module coherence = network hub (Zi > 2.5, Pi > 0.62); and (v) peripherals—for nodes within the module but few outside connection (Zi < 2.5 and Pi < 0.62; Olesen et al., 2007). The network data were obtained from the online platform MENA and was rearranged and analysed with the open‐source software Cytoscape (V3.0.X; Shannon et al., 2003). Network was filtered (degree between 5 and 81 inclusive) before cluster analysis. Clustering was obtained with plugin CytoCluster (V2.1.0; M. Li et al., 2017), an app for analysis and visualization of clusters from network. We used OH‐PIN algorithm with default values for clustering. Clusters were manually evaluated to determine dominant taxa.

## RESULTS

### Data structure

We sequenced 19 samples in total and obtained 91,000–118,000 reads per sample, of which 60% passed quality filtering. 98% of the filtered reads were above the Q20 threshold (Table S1). We found a minimum of 1244 OTUs and a maximum of 2193 OTUs per sample (average 1835 OTUs per sample); total reads per sample were on average 73,262, of which approx. 15% were unique OTUs (Figure S2). The rarefaction curves showed that sequencing depth was adequate for describing the microbial communities in our samples (Figure S3A). The curves became flatter, even if we grouped the samples into origin classes (F = fruiting body, M = mycelium mats, WS = wooden substrate and MS = mineral substrate) and hence just scarce species remain to be elusive (Figure S3B).

### Bacterial abundance and community composition of fungal material and surrounding

OTUs were used to analyse differences in biodiversity and abundance of fungal fruiting bodies, mycelium mats and substrate samples (wooden and mineral substrate). Bacteria were identified as the most diverse and abundant group of prokaryotes; archaea were found in lesser abundances and lower diversity (Table S2 and Figure S4). One mineral substrate and mycelium sample (Tarrenz) had 39% and 49%, respectively, exceptionally high numbers of archaea OTUs (e.g., Halobacterota); in all other samples, archaeal OTUs accounted for 0.09%–7% of all OTUs. The most abundant bacterial phyla across all samples were Actinomycetota and Pseudomonadota (mean 44.63% and 33.36%), followed by Bacteroidota and Bacillota (7.9% and 4.66%), although again the sample from Tarrenz was a clear outlier (e.g., 30% of fruiting body or 27% of wood community was assigned to Bacteroidota, while 7.9% was the average). Other abundant phyla were Acidobacteriodota (G.FB1 with 14% and SM.WS with 19%) and Chloroflexota (WE.FB2 with 13% and WE.MS with 15%; Table S2).

**FIGURE 2:**
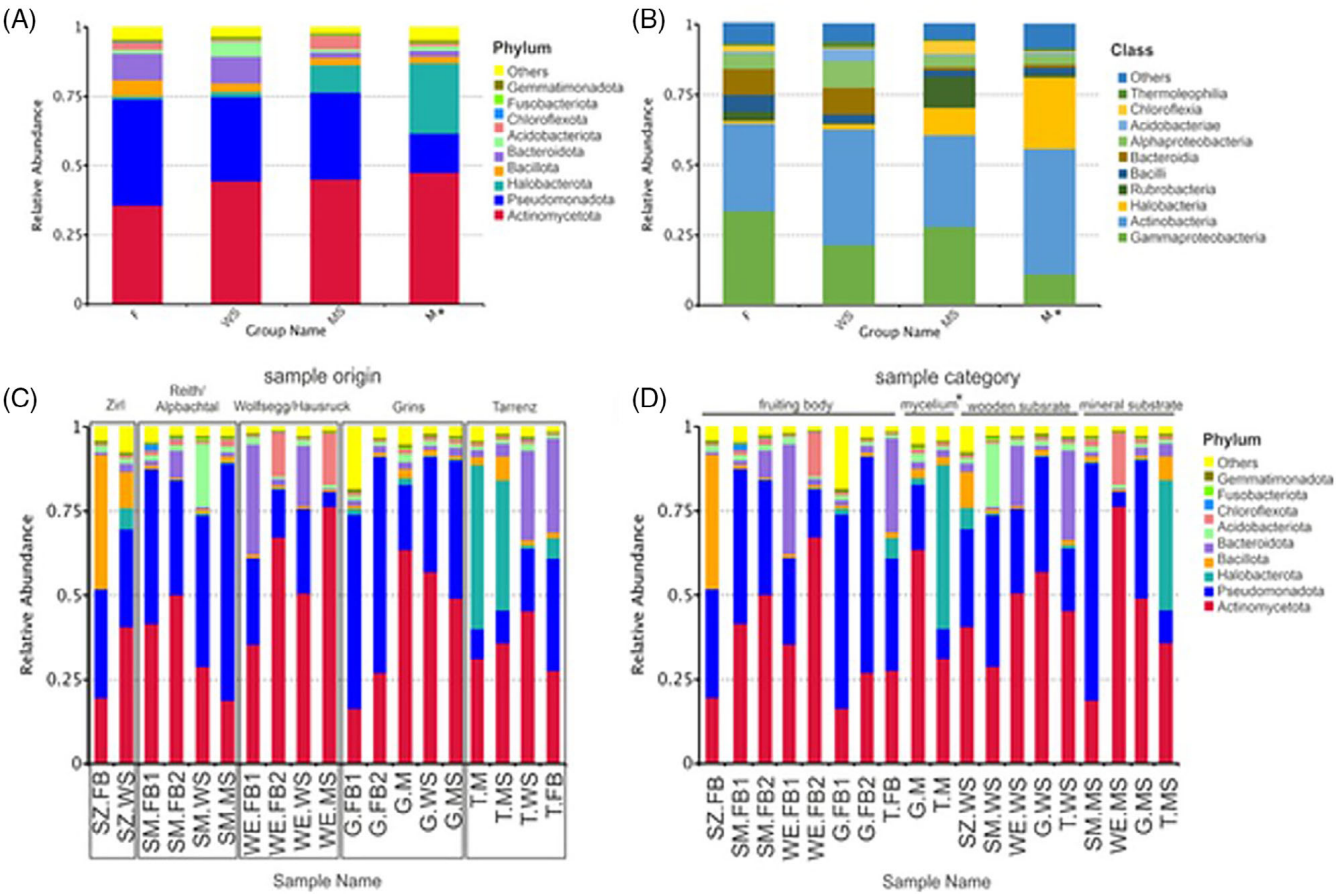
Prokaryote biodiversity and abundance are different in fungal fruiting bodies, mycelium mats, wooden substrate and mineral substrate. The mean relative abundance (%) of 10 most numerous bacterial (A) phyla, (B) classes. (A + B) based on sample type: F (fruiting body), WS (wooden substrate), MS (mineral substrate) and M (mycelium mats). (C + D) The mean relative abundance (%) of the 10 most numerous bacterial phyla in the individual samples (C) grouped based on sample origin and (D) grouped based on sample category (fruiting body, mycelium mats, wooden, and mineral substrate). *M (mycelium mats sample) *n* = 2, samples were included to display a trend. Graph is based on read numbers normalized by the total number of reads per sample.

The most abundant bacterial phylum associated with *S. lacrymans* fruiting bodies was Pseudomonadota (38.4%), followed by Actinomycetota and Bacteroidota (35.7% and 5.9%). The mycelium mats were dominated by OTUs belonging to the Actinomycetota (47.5%), Halobacterota (25.3%), and Pseudomonadota (14.1%). Actinomycetota was the most abundant phylum in both environment samples (WS—44.5% and MS—45.1%), followed by Bacillota in wood (31.6%) and Pseudomonadota in mineral substrate samples (31.4%). With 30.4%, Pseudomonadota were abundant in wood. Other prominent phyla were Bacteroidota in wooden substrate (9.8%) and Halobacterota in mineral substrate material (9.9%) (Figure 2A). On class level *Gammaproteobacteria* and *Actinobacteria* were found to dominate in fruiting bodies (33% and 30.8%), whereby *Actinobacteria* was most abundant on mycelium mats and the environmental samples (M—44.5%, WS—41.2% and MS—32.4%).

The prokaryotic community of woody and mineral substrates included *Gammaproteobacteria* (WS—20.9% and MS—27.4%). *Gammaproteobacteria* were also found in mycelium mats, but were represented by fewer reads (10.7%). Halobacteria were found associated with mycelium samples (25.3%) and mineral substrate material (9.9%). Another abundant class of prokaryotes in mineral substrate was Rubrobacteria (10.8%; Figure 2B).

**FIGURE 3:**
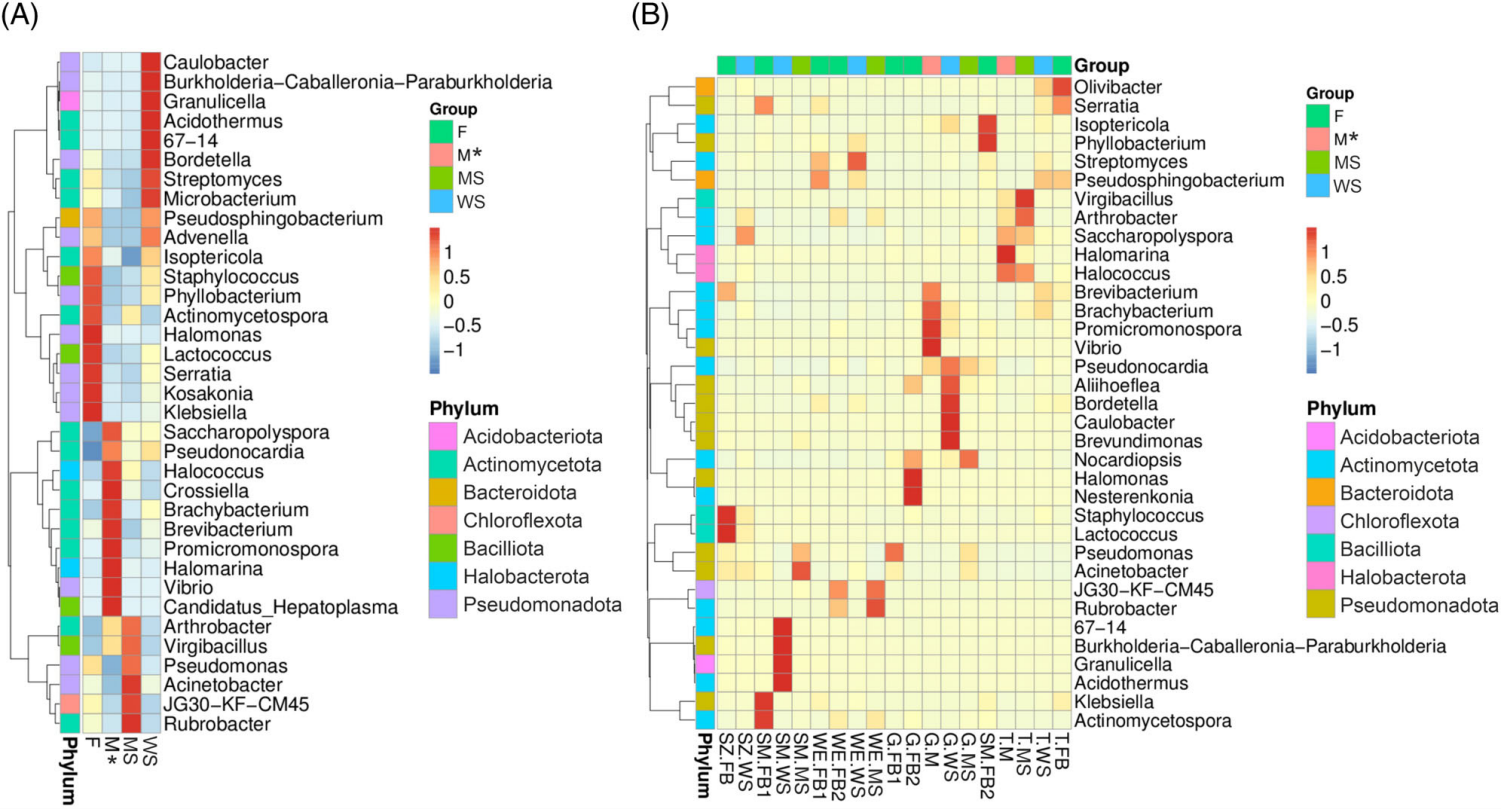
Taxonomic abundance cluster heat map (top 35 genera across samples) showing that prokaryote communities associated with different samples (mycelia, fruiting bodies, wood and mineral substrate) form distinct clusters. (A) Assignment of samples to groups (F = fruiting body, M = mycelium mats, MS = mineral substrate material and WS = wood substrate), cumulative application. (B) Illustration of individual samples. The *Y*‐axis represents the genus and the absolute value of ‘*z*’ (see colour scheme red to blue) represents the distance between the raw score and the mean of the standard deviation. ‘*Z*’ is negative when the raw score is below the mean, and vice versa. Application by abundance. *M (mycelium mats sample) *n* = 2, samples were included for sake of completeness.

Plotting of the 35 most abundant genera into a heatmap confirmed the trend visible in Figure 2C,D, that is, that there are distinct bacterial genera clusters for each sample type (Figure 3A) and the clusters are about the same size (F—11, M—10 and WS—10 members), except the cluster for mineral substrates, where just six members were detected. The bacterial community of the fruiting body was dominated by OTUs belonging to the phylum Pseudomonadota (55%), while Actinomycetota were dominant in samples derived from the mycelial mats (60%). Actinomycetota and Pseudomonadota emerged as abundant in mineral substrates and mycelial mats (33% and 40%). The genera *Advenella* sp. and *Pseudosphingobacterium* sp. were found to be similarly abundant in fruiting body and wood samples. Illustration of each individual sample shows clustering of samples from the same location, but also highlights pattern across sample types (Figure 3B).

We used ternary plots to further screen for dominant taxa among the sample groups (fruiting body, wood and mineral substrate). Genera, such as *Pseudomonas*, *Acinetobacter* and *Pseudonocardia*, were evenly distributed over sample groups, while *Rubrobacter* and *Halococcus* were preferably found associated with the mineral substrate. Bacteria from the *Burkholderia‐Caballeronia‐Paraburkholderia* complex were identified on wood, while *Halomonas* sp. was found for fruiting body (Figures S5 and S6). When the data set of the ternary plots was subdivided according to sampling locations, genera patterns shift, that is, for Grins, *Pseudonocardia* and *Pseudomonas* were more abundant compared to samples from Tarrenz or Wolfsegg/Hausruck (although they were not significantly assigned to one tissue/sample type; Figure S7A). Otherwise, the *Burkholderia‐Caballeronia‐Paraburkholderia* complex was found to be allocated to wood and *Acinetobacter* sp. and *Pseudomonas* sp. to mineral substrate from Reith/Alpbachtal (SM; Figure S7B). The material of the mineral substrate from Tarrenz was dominated by *Halococcus* sp.; another abundant genus was *Pseudosphingobacterium* sp. that was equally distributed between fruiting bodies and wood material (Figure S7C). We found *Streptomyces* and *Pseudosphingobacterium* sp. as genera with major abundance in samples from Wolfsegg/Hausruck. *Rubrobacter* sp. was found associated with mineral substrate (Figure S7D).

### Microbial diversity

**FIGURE 4:**
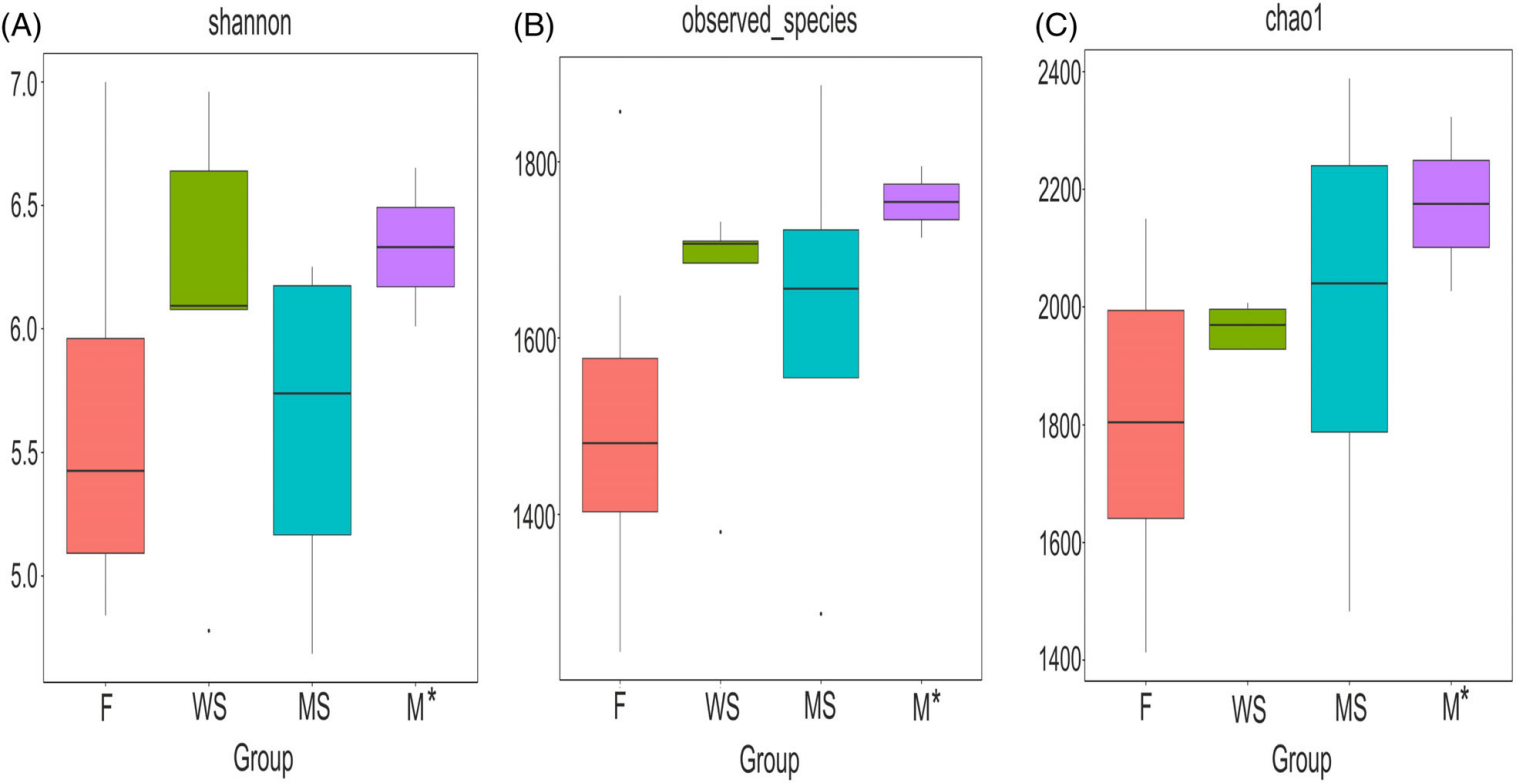
Alpha‐diversity indices are more similar between mycelium mats and mineral/wood substrate samples than between samples from mycelium mats and fruiting body. Alpha diversity boxplots for differences in (A) Shannon Indexes, (B) observed species and (C) Chao1 richness index. Fruiting body (F in salmon‐coloured), woody material (WS in green), mineral substrate samples (MS in blue) and fungal mycelium mats (M in turquoise). M (mycelium mats) *n = 2, samples were included for sake of completeness.

**FIGURE 5:**
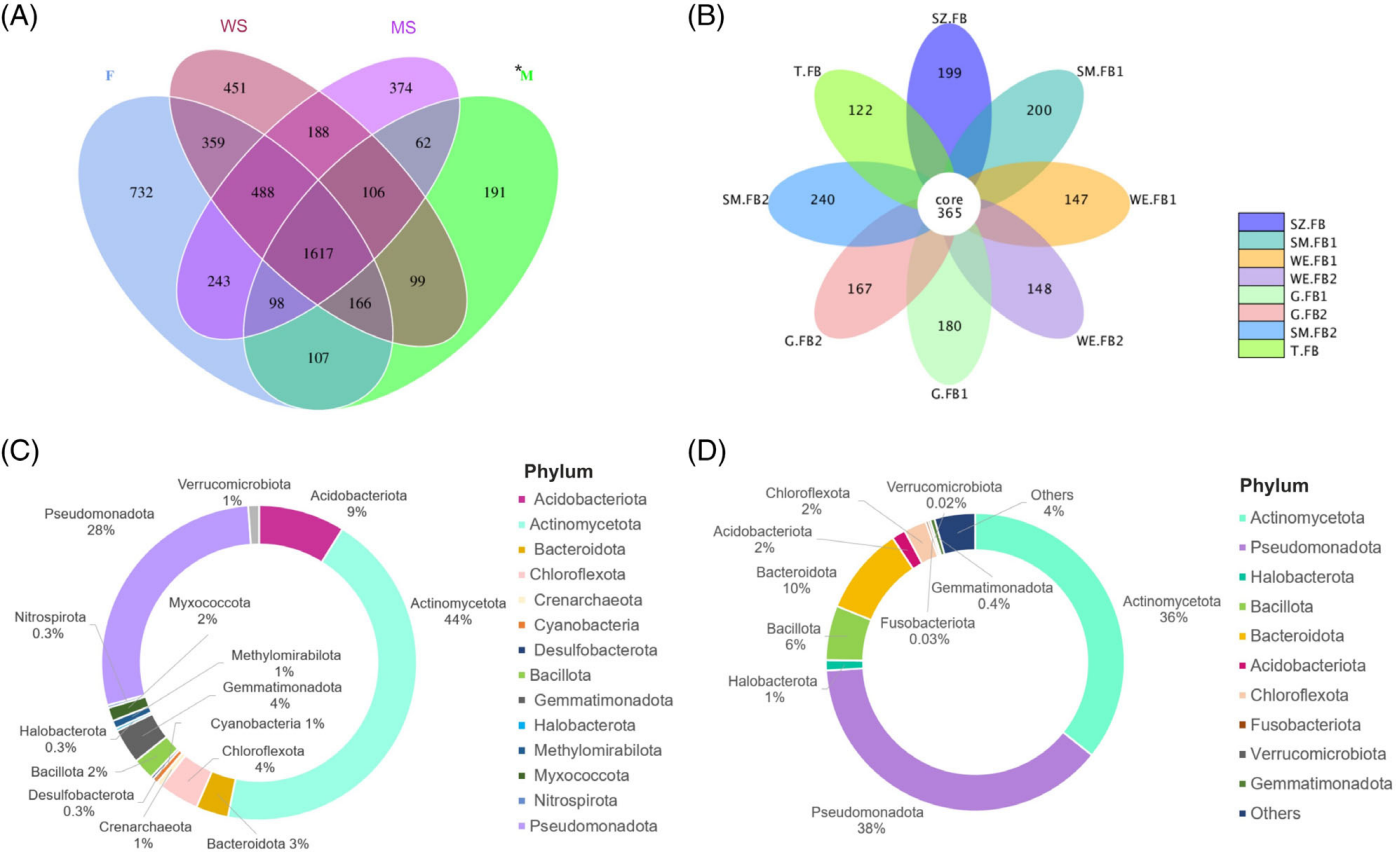
Thirty per cent of OTUs were shared across all samples and Actinomycetota dominated the core microbiome of *Serpula lacrymans* fruiting bodies. The numbers of unique and shared OTUs in the fruiting bodies (F), mycelium mats (M), and in the surrounding wood (WS) and mineral substrate (MS). (A) Venn diagram, (B) flower Diagram, (C) Core microbiome of *S. lacrymans* fruiting bodies and (D) relative abundance of top 10 phyla associated with fruiting bodies of *S. lacrymans*. Different phyla are indicated with colour code (legend right side). M (mycelium sample) *n* = 2, samples were included for sake of completeness. OTU, operational taxonomic unit.

Alpha diversity indices, including observed species, Chao1, Shannon index, phylogenetic diversity (PD) whole tree and abundance‐based coverage estimator (ACE), were calculated (Table S3). Chao‐1 is a measure of total richness and is particularly useful because of a valid variance that is used to calculate confidence intervals (Chao, 1987; M. Wang et al., 2005). The Shannon index reflects species numbers and evenness of species abundance (Guinane et al., 2013). Our results showed that the bacterial diversity indices were the highest for samples from mycelium mats (Figure 4A–C), but these values should be interpreted with caution as only two distinct samples could be analysed. Prokaryote communities associated with fruiting bodies were less diverse than those from other samples; however, these differences were not significant (Table S4). Overall, 1617 (30.6%) bacterial OTUs were found in all samples, while 732 OTUs (13%) were only present in samples from fruiting bodies, 451 OTUs in wood samples (8.5%) only, 374 OTUs solely in mineral substrates (7.1%), and 191 OTUs exclusively in the mycelial mats (3.6%; Figure 5A).

The core microbiome of *S. lacrymans* fruiting bodies analysed in this study comprised 365 OTUs (Figure 5B), while on average, 175 unique OTUs were found per fruiting body. The fruiting body core microbiome was dominated by the genera *Nocardioides*, *Pseudomonas*, *Pseudonochardia*, *Streptomyces* and *Rubrobacter*. Rarer core OTUs belonged to *Solibacter*, *Saccharopolyspora*, *Vicinamibacteraceae*, *Sphingomonas* and *Acinetobacter* (see Supplement Table 01_coreFB).

Core OTU numbers were dominated by Actinomycetota, as 160 (44%) of the 365 OTUs were assigned to this phylum. Another phylum with high OTU counts was Pseudomonadota (28%), followed by Acidobacteriota (9%), Chloroflexota, Gemmatimonadota (both 4%), Bacteriodota (3%) and Bacillota (2%; Figure 5C). Interestingly, core OTUs of fungal fruiting bodies included phyla from the domain Archaea, although these had very limited abundance (Crenarchaeota—0.5% and Halobacterota—0.3%). In comparison, the fruiting body pan microbiome was dominated by Pseudomonadota and Actinomycetota (38% and 36%), followed by Bacteriodota (10%) and Bacillota (6%; Figure 5D).

Beta diversity was analysed with weighted‐UniFrac analysis (Figure S8A,B; F—MS *p* = 0.95/ F—WS *p* = 0.64/ MS—WS *p* = 0.69 by Wilcox‐test). Although the differences in communities between distinct samples emerged as not significant, the β‐diversity of the fruiting body sample from Grins (G.FB1) and mycelial and samples from the mineral substrate in Tarrenz (T.M and T.MS) was higher compared to other samples (Figure S8A). Beta diversity of fruiting bodies was more similar to the one of wood samples than that of samples from the mineral substrate. UniFrac‐based PCoA provided an entire comparison of microbial communities and showed that the most distinct communities of wood material and samples from the mineral substrates were both more similar to communities of fruiting bodies than to each other. Communities of mycelial mats were more different to communities of other samples (Figure S8C, caution *n* = 2).

### Network analysis

**FIGURE 6:**
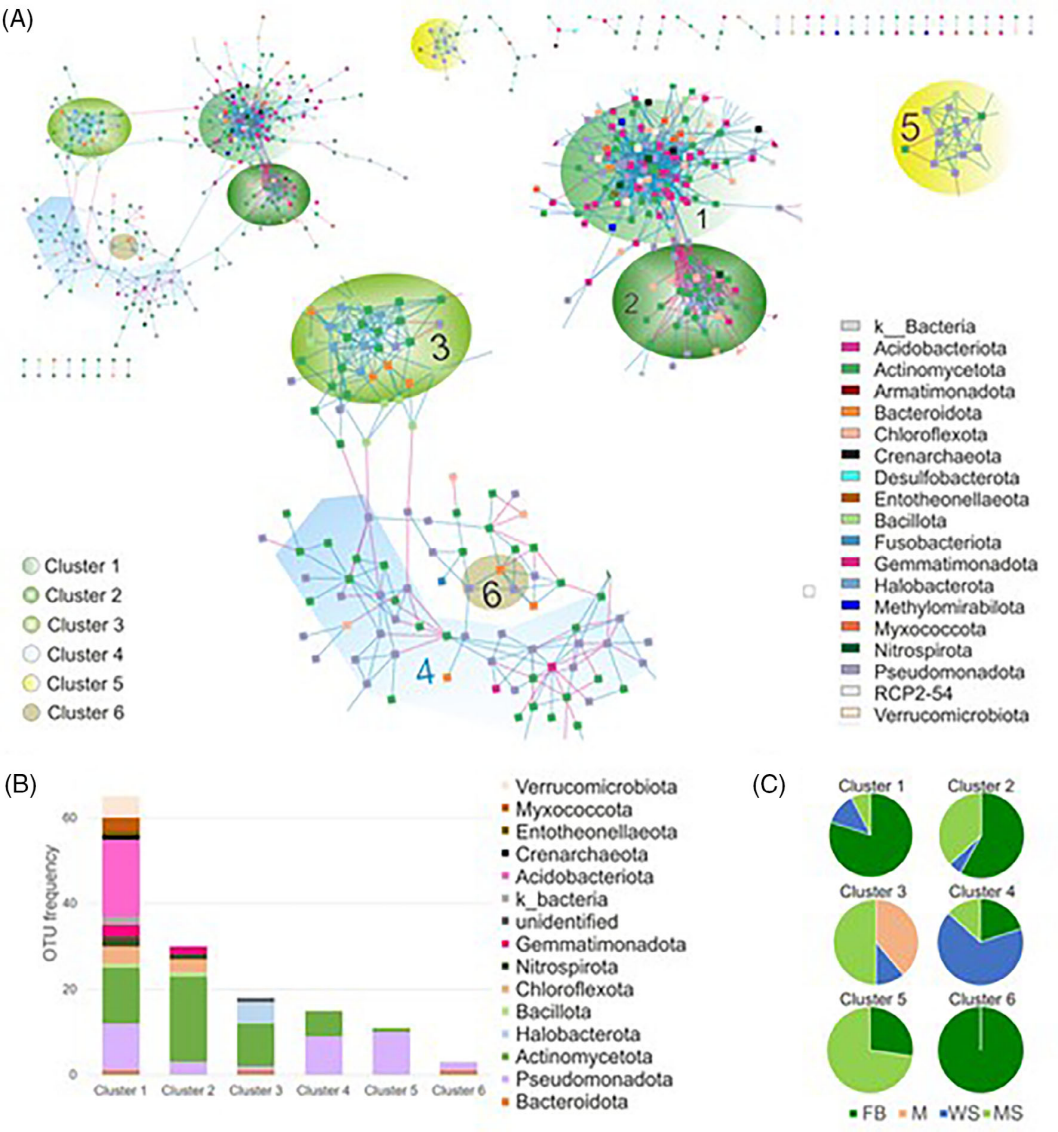
Microbial co‐occurrence network showing clusters for distinct sample types. Each node (vertex) indicates a single OTU at 97% sequence similarity and different colours distinguish the nodes (i.e., OTUs) at the phylum level. (A) Network showing co‐occurrence of OTUs, annotation to phyla (legend right bottom corner) and clustering. Left upper corner shows the entire network (415 nodes and 1141 edges); in the middle part, an enlargement with clusters is displayed (149 nodes and 764 edges). Positive links are in blue and negative links are in red. (B) Bar plot showing OTU frequency (OTU richness) and taxonomic composition of clusters, and (C) Structure of clusters considering the main source of the OTUs. Fruiting bodies (F), mycelial mats (M) and in the surrounding wood (WS) and mineral substrates (MS). Note for mycelium *n* = 2. For global network properties, see Table S5. OTU, operational taxonomic unit.

An ecological network was constructed using the bacterial OTUs from the 19 dry rot‐associated samples. This analysis resulted in a clustered network containing 415 nodes and 1141 edges (i.e., links; Figure 6A). The similarity threshold of the network was 0.76. Other indices, such as average connectivity, average path length, average clustering coefficient and modularity are summarized in Table S5. The network showed positive or negative interactions among taxa, whereby 184 out of 1141 (16%) interactions were negative and 957 (84%) were positive (Table S5). A total of six clusters were identified, containing about 34% of the total OTUs: cluster 1 contained 65 OTUs, cluster 2 contained 30 OTUs, cluster 3 contained 18 OTUs, cluster 15 contained 6 OTUs, cluster 5 contained 11 OTUs, and cluster 6 contained three OTUs. Taxonomic classifications at phylum level in the network are shown in Figure 6B. Generally, Actinomycetota, Pseudomonadota and Acidobacteriota were most abundant in the clusters, which is in accordance with their generally high abundance. Archaeal OTUs of the phyla Thermoproteota and Halobacterota were as well present in the network, although in minor amounts.

Six OTUs were identified as cluster hubs, that is, highly connected nodes within clusters with Zi > 2.5 (within‐module connectivity (Zi) = closeness centrality), namely OTUs number 70 (Nitrospirota, *Nitrospira* sp.), 173 (Actinomycetota, order Gaiellales), 249 (Acidobacteriota, candidatus *Solibacter* sp.), 361 (Acidobacteriota, order Acidobacteriales), 390 (Actinomycetota, order Gaiellales) and 2822 (Pseudomonadota, family Xanthobacteraceae). All of them participate in cluster 1, while OTU173 is as well found in cluster 2. It was further analysed if OTUs from clusters are predominantly occurring within a distinct sample (Figure 6C). Clusters 1, 2 and 6 were predominantly comprised of OTUs mainly found associated with fruiting bodies (80%, 58% and 100%), while cluster 4 was dominated by OTUs found mainly in wood (67%). Clusters 3 and 5 were predominantly comprised of OTUs mainly identified in underground material (50% and 73% respectively). OTUs associated mainly with mycelium were exclusively found in cluster 3.

## DISCUSSION

Using an amplicon‐based approach, the prokaryote communities associated with *S. lacrymans* were found to form tissue‐ and substrate‐dependent clusters. The core community across all sample types was quite large with one third of the OTUs identified in all samples. The number of unique OTUs was small. Fruiting bodies of *S. lacrymans* shared a core set of 365 OTUs, dominated by Actinomycetota, Pseudomonadota and Acidobacteriodota. Tissue/sample type emerged as the main driver for microbial diversity, followed by sample origin (Figure 3). *S. lacrymans* forms resupinate fruiting bodies (which are adjacent to the substrate); hence, it can be expected that the fruiting body community is in bigger parts influenced by the community of the substrate on which the fruiting body has developed. Previous culture‐based analyses of different *S. lacrymans* tissue types using a highly similar sampling approach have revealed similarly marked differences: fruiting bodies and mycelia were dominated by Bacillota (formerly ‘*Firmicutes*’), rhizomorphs were dominated by Pseudomonadota (*Proteobacteria*), and in both tissues Actinomycetota and Bacteroidota (‘*Actinobacteria*’ and ‘*Bacteroidetes*’) were present but less abundant (Embacher et al., 2021). Using amplicon sequencing, OTUs belonging to the same phyla were detected; however, their abundances were different compared to the culture‐based studies. These differences are partly the result of the fundamentally different methodological approaches, as culture‐based approaches favour different taxa compared to DNA‐based approaches (Embacher et al., 2021; Fonseca, 2018). Also, our amplicon approach did not address the issue of non‐viable prokaryotes or exogenous DNA (Probst et al., 2021).

Nonetheless, amplicon sequencing is able to gain insight into the abundance and structure of prokaryote communities. Combining the findings from amplicon‐based diversity analyses and culture‐based approaches allows to identify the most abundant bacterial phyla in fungal tissue and its surroundings and to get a first idea about the recruitment and specificity of selected taxa. Fruiting bodies showed the highest abundance (and diversity) of Pseudomonadota at the OTU level, while in culture‐based studies, Bacillota were the most abundant group (Embacher et al., 2021). In mycelia, OTUs of Actinomycetota had a high abundance, while Bacillota dominated culture‐based approaches. Culture‐based experiments revealed that *Kluyvera* spp. (Pseudomonadota), *Paenisporosarcina* spp., *Bacillus* spp. and *Staphylococcus* spp. (all Bacillota) were most abundant on fruiting bodies of *S. lacrymans*. Another common genus was *Flavobacterium* spp. (Bacteriodetes). Some of these species which were abundant in culture‐dependent approaches form endospores (e.g., *Bacillus* spp.), so it cannot be excluded that the high abundance of these species is biased by a stochastic ‘mixing’ and not a ‘true’ interaction. As OTU abundance and prevalence of taxa previously identified as dominating fruiting bodies were upstaged with amplicon sequencing, this hypothesis appears more likely (see Supplement Table 02_Top35taxa). This reinforces that especially Bacillota, which were the most abundant endospore‐forming bacteria in isolation‐based approaches, are likely not true interaction partners of *S. lacrymans*.

Distinct differences in the diversity and abundance of prokaryote taxa were found in the different sample types. OTU‐based clusters from fruiting bodies clearly separated from the mycelium, and vice versa. The biodiversity pattern from fungal tissue was more similar to the corresponding substrates than to each other, indicating that part of the *S. lacrymans* microbiome is recruited from the environment by stochastic processes (Figure 3). An overlap in abundance of *Advenella* sp. and *Pseudosphingobacterium* sp. for fruiting body and wood samples was detected and some genera typically found associated with mineral substrates were as well present in fruiting bodies (e.g., *Pseudomonas* sp. or *Actinomycetospora* sp.). This indicates that bacteria are migrating from the substrate into fungal tissues and in developing fruiting bodies of *S. lacrymans* where only some selected species are able to persist. If this recruitment is the result of a random, stochastic event (Kielak et al., 2016) or if there is an environmental co‐selection (Deveau et al., 2018; Johnston et al., 2018) or if this is part of a specialized interaction (Boersma et al., 2009; Christofides et al., 2019) remains to be investigated. The prokaryotic biodiversity found associated with the two samples from mycelial mats analysed in this study was more dissimilar to the biodiversity associated with fruiting bodies than with the biodiversity of the corresponding substrates (Figure 3). In contrast, alpha‐diversity indices were more similar between mycelium mats and substrates than between mycelium mats and fruiting bodies. However, due to the small sample size for mycelial mats artefacts cannot be excluded, so more samples will be needed to draw robust conclusions.

The main link between OTUs in the clusters of the network was sample type, as 80% of OTUs found for cluster one are associated with fruiting bodies, and other clusters by OTUs associated with wood or mineral substrate (Figure 6C). We found negative feedbacks between the clusters containing mainly substrate‐based OTUs (Clusters 3 and 4), while those clusters were linked only by single (mainly positive) links to the clusters dominated by fruiting‐body derived OTUs (Figure 6). In other studies investigating fruiting body‐associated species, the relative abundance of bacterial groups varied between different parts of the fruiting bodies (e.g., pileus and stipe of *Morchella sextelata* or *Tricholoma matsutake*) and these communities differed from soil underneath the mushrooms (Benucci et al., 2019; Oh et al., 2018). As already mentioned, a comparison of these findings with those from resupinate fruiting bodies is limited. There is no differentiation between stipe and cap and the contact zone with the substrate embraces the lower surface of the entire fruiting body. Nonetheless, different fungal parts differ in tissue architecture and chemical composition, which could be responsible for the presence of different prokaryotic communities on the respective fungal tissue (Deja et al., 2014). The main selectors for microbial diversity associated with *S. lacrymans* samples emerged to be sample type, while the influence of the geographical origin of the samples seemed to be second to that. Similar findings have been reported for other fungi where climate and soil factors were negligible in comparison to host phylogeny (Gohar et al., 2022). However, other studies argue that soil physical and chemical characteristics are important for the structure of mushroom‐inhabiting bacterial communities at local scales (Pent et al., 2017, 2020; Splivallo et al., 2019), which was likely the main driver for the differing diversity found in samples from different origin. To identify the main drivers that select for the mycobiome, more controlled samplings of the environment will need to be conducted (e.g., type and age of substrate, pH and humidity). But the presented data support that *S. lacrymans* itself has a core microbiome associated with different tissue types, while an extensive accessory microbiome is the result of stochastic selection from the environment.

All samples analysed shared 30% of their OTUs. The diversity of OTUs in fruiting bodies was lower than for the other samples. Interestingly, the number of unique total OTUs was highest in fruiting bodies. In previous culture‐based studies, fruiting bodies had the lowest bacterial colony‐forming units per g (CFU g^−1^) tissue, but the highest phylogenetic diversity (Embacher et al., 2021). We detected 365 OTUs as core microbiome of all *S. lacrymans* fruiting bodies. Those OTUs mainly belonged to the Actinomycetota and Pseudomonadota, followed by Acidobacteriodota. The identified *S. lacrymans* basidiomata core microbiome is very similar to the genera described to be the core global mushroom microbiome (Gohar et al., 2022). The core mushroom microbiome comprises the genera *Halomonas*, *Serratia*, *Bacillus*, *Cutibacterium*, *Bradyrhizobium* and *Burkholderia* (Gohar et al., 2022), all of which, except *Bradyrhizobiuim*, were also detected in the core microbiome of fruiting bodies of *S. lacrymans*. Especially OTUs belonging to *Serratia* and *Halomonas* were abundant, while OTUs belonging to *Bacillus*, *Burkholderia* and *Cutibacterium* were rare. Archaea OTUs (Crenarchaeota and Halobacterota) were also part of the fruiting body core microbiome, similar to findings in forest mushrooms (Rinta‐Kanto et al., 2018). The five most abundant phyla in the fruiting body core microbiome were Actinomycetota (44%), Pseudomonadota (28%), Acidobacteriota (9%), Chloroflexota (4%) and Gemmatimonadota (3.6%), while the most abundant phyla in the pan microbiome were Pseudomonadota and Actinomycetota (38% and 36%), followed by Bacteriodota (10%) and Bacillota (6%). In comparison to the core, the abundance of Acidobacteriota, Gemmatimonadota and Chloroflexota diminished in the pan microbiome, while Bacillota and Bacteriodota were enriched, which could hint for a shift of secondary microbiota to primary microbiota.

The revealed fruiting body core microbiome comprised cellulolytic and xylanolytic *Pseudonocardia* (Kaewkla & Franco, 2021; Malfait et al., 1984), *Saccharopolyspora* spp. that produces xylanase (Sinma et al., 2011), glycosyl transferases (Schmidt‐Bohli, 2016), cellulase (Meena et al., 2013), and amylases (Chakraborty et al., 2011; Xu et al., 2016), or *Sphingomonas* spp., which were shown to have an increased potential to use complex carbohydrates including cellulose, chitin and hemicellulose (Tláskal & Baldrian, 2021). Other species were *Pseudomonas* spp. and *Acinetobacter* spp., taxa which are known for their nitrogen‐fixing abilities (Liba et al., 2006; M. Wang et al., 2020; Yaghoubi Khanghahi et al., 2021). Burkholderiales, a class for which a mycophagous lifestyle was suggested (Brabcová et al., 2018; Hervé et al., 2016; Starke et al., 2020; Valášková et al., 2009), was as well quite abundant. Recent studies have identified distinct strategies of bacteria to fulfil nutritional aspects in deadwood and soil: (i) ‘decomposer’ species with high potential to degrade complex C‐substrates (mainly bacteria from Acidobacteriota and Bacteroidota) and (ii) more ‘opportunistic’ bacteria that depend on labile or alternative carbon sources (mycophagy; e.g., Pseudomonadota; Lladó et al., 2019; Tláskal & Baldrian, 2021). The latter were found to be more active in N‐cycling, thereby providing a valuable task for the whole community (e.g., via N_2_ fixation; Tláskal & Baldrian, 2021). Nonetheless, the physiological abilities of prokaryotes associated with *S. lacrymans* remain as open question and further research is needed to assess if and to what extent the prokaryotes detected in this study belong to the proposed groups of ‘opportunistic’ or ‘decomposer’ bacteria.

## CONCLUSION

This study provides further insights into the prokaryotic community associated with *S. lacrymans* and reveals that there is a core community associated with fungal fruiting bodies that is dominated by Actinomycetota and Pseudomonadota. Increasing information on deadwood bacteriomes and specific fungus‐influencing microbiota hint at the importance of distinct microbes during wood decay, which potentially fulfil a precisely regulated set of tasks that help and influence others. In the case of timber decaying *S. lacrymans*, the exact succession and collaboration mechanisms (i.e., which taxa are helpful for wood decay of *S. lacrymans* and what is the underlying machinery?) are for the most part elusive. Here, we delivered further evidence of which species are associated with fruiting bodies of *S. lacrymans* and showed that the bacterial community of the ambient environment is distinct from that of the fungal tissue. As different fungal developmental stages or tissues have a distinct physiological set‐up and function, it is plausible that each phenotype could have specific microbial partners. Our results support this scenario as bacterial genera cluster are separated between fruiting bodies and mycelium. Additionally, assessment of the physiological properties of bacteria that are useful in the fungus/wood environment (i.e., cellulolytic power, mycophagic behaviour or involvement in nitrogen cycling) leave space for further research as bacteria from Actinomycetota are known for their ligninolytic abilities (Leo et al., 2018). Another recent publication assumed that phylogenetically different fungal hosts that fill the same ecological niche, harbour closely related bacterial taxa, and proposed that bacteria might prefer fungi with selected trophic modes (Robinson et al., 2021). Therefore, our study provides the first puzzle pieces to resolve if fungi occupying and decaying indoor wood provide niches for closely related bacterial taxa, although further studies with fungi of similar trophic modes are necessary.

## AUTHOR CONTRIBUTIONS

**Julia Embacher:** Conceptualization (equal); data curation (lead); formal analysis (lead); funding acquisition (equal); investigation (lead); methodology (lead); visualization (equal); writing – original draft (equal); writing – review and editing (equal). **Susanne Zeilinger:** Conceptualization (equal); project administration (equal); resources (equal); supervision (equal); writing – original draft (equal); writing – review and editing (equal). **Martin Kirchmair:** Conceptualization (equal); data curation (equal); funding acquisition (equal); methodology (equal); resources (equal); supervision (equal); writing – original draft (equal); writing – review and editing (equal). **Sigrid Neuhauser:** Conceptualization (equal); data curation (equal); formal analysis (equal); funding acquisition (equal); project administration (equal); resources (equal); supervision (equal); writing – original draft (equal); writing – review and editing (equal).

## CONFLICT OF INTEREST STATEMENT

The authors declare no conflicts of interest.

## Supporting information


**DATA S1.** Supporting Information.

**Supplement Table 01_coreFB:** Depicting core FB values.

**Supplement Table 02_Top35taxa:** Depicting values of top 35 taxa.

## Data Availability

The sequences obtained from each sample were submitted to Sequence Read Archive and are available under BioProject PRJNA878418 (accession numbers SAMN30726529 to SAMN30726547).
